# Hemodynamic assessment of critically ill patients using a miniaturized transesophageal echocardiography probe

**DOI:** 10.1186/cc12793

**Published:** 2013-03-27

**Authors:** Luca Cioccari, Hans-Rudolf Baur, David Berger, Jan Wiegand, Jukka Takala, Tobias M Merz

**Affiliations:** 1 Department of Intensive Care Medicine, Bern University Hospital (Inselspital) and University of Bern, Bern, Switzerland; 2 Herzklinik Bern, Spital Sonnenhof, Buchserstrasse 30, 3006 Bern, Switzerland

**Keywords:** hemodynamic monitoring, trans-esophageal echocardiography, quality assessment, critical care

## Abstract

**Introduction:**

Hemodynamic management in intensive care patients guided by blood pressure and flow measurements often do not sufficiently reveal common hemodynamic problems. Trans-esophageal echocardiography (TEE) allows for direct measurement of cardiac volumes and function. A new miniaturized probe for TEE (mTEE) potentially provides a rapid and simplified approach to monitor cardiac function. The aim of the study was to assess the feasibility of hemodynamic monitoring using mTEE in critically ill patients after a brief operator training period.

**Methods:**

In the context of the introduction of mTEE in a large ICU, 14 ICU staff specialists with no previous TEE experience received six hours of training as mTEE operators. The feasibility of mTEE and the quality of the obtained hemodynamic information were assessed. Three standard views were acquired in hemodynamically unstable patients: 1) for assessment of left ventricular function (LV) fractional area change (FAC) was obtained from a trans-gastric mid-esophageal short axis view, 2) right ventricular (RV) size was obtained from mid-esophageal four chamber view, and 3) superior vena cava collapsibility for detection of hypovolemia was assessed from mid-esophageal ascending aortic short axis view. Off-line blinded assessment by an expert cardiologist was considered as a reference. Inter-rater agreement was assessed using Chi-square tests or correlation analysis as appropriate.

**Results:**

In 55 patients, 148 mTEE examinations were performed. Acquisition of loops in sufficient quality was possible in 110 examinations for trans-gastric mid-esophageal short axis, 118 examinations for mid-esophageal four chamber and 125 examinations for mid-esophageal ascending aortic short axis view. Inter-rater agreement (Kappa) between ICU mTEE operators and the reference was 0.62 for estimates of LV function, 0.65 for RV dilatation, 0.76 for hypovolemia and 0.77 for occurrence of pericardial effusion (all *P *< 0.0001). There was a significant correlation between the FAC measured by ICU operators and the reference (*r *= 0.794, *P *(one-tailed) < 0.0001).

**Conclusions:**

Echocardiographic examinations using mTEE after brief bed-side training were feasible and of sufficient quality in a majority of examined ICU patients with good inter-rater reliability between mTEE operators and an expert cardiologist. Further studies are required to assess the impact of hemodynamic monitoring by mTEE on relevant patient outcomes.

## Background

Hemodynamic monitoring plays an important role in the management of intensive care unit (ICU) patients. Monitoring using a pulmonary artery catheter has been questioned due to the invasive nature of the method and the lack of clear evidence for improved outcomes associated with its insertion and use to guide therapy [1]. Several alternative monitoring technologies have been introduced for the monitoring of cardiac output and stroke volume or their surrogates, such as trans-pulmonary thermodilution and various other less invasive techniques. However, the value of these monitoring tools also remains controversial and randomized controlled studies showing improvement of relevant patient outcome parameters due to the use of these devices do not exist [2]. Decreased accuracy under certain conditions, complexity of use and interpretation, and the need for specially trained staff are common to all available monitoring tools [3].

Echocardiography has been established as a tool to evaluate the causes of hemodynamic instability in ICU patients by the visualization of cardiac chambers, valves and pericardium and cardiac functional abnormalities [4,5]. Transthoracic echocardiography (TTE) can be used as a first-line approach for a quick and focused examination to diagnose acute cor pulmonale, cardiac tamponade or major left ventricular systolic dysfunction [6]. The training necessary to reliably perform such an abbreviated TTE use is substantial [7] and the method is not readily available for every intensivist. Trans-esophageal echocardiography (TEE) can have a better diagnostic capability and is more reproducible than TTE [8]. A minimum number of 31 TEE examinations has been reported to be required for intensivists to achieve competence in TEE driven hemodynamic evaluation of ventilated ICU patients [9]. Additionally, repeatedly inserting the TEE probe as required for serial evaluation of a patient's hemodynamic status is associated with a small but significant risk of injury to oral and esophageal structures [6].

Recently, a new technology using miniaturized probes for continual or repeated prolonged monitoring using TEE (mTEE) has been introduced. This approach potentially provides a robust, but more rapid and user-friendly approach to monitoring hemodynamic status and cardiac function than conventional TTE and TEE. In a recently published study by Vieillard-Baron *et al*. the feasibility of hemodynamic monitoring and safety of mTEE were demonstrated in a group of 94 ventilated critically ill patients [10]. In this study, mTEE examinations were performed by four highly trained intensivist with extensive expertise in critical care echocardiography. However, the results of this study cannot be extended to prove the ability of operators with no previous experience in TEE to efficiently conduct hemodynamic assessment using mTEE. The usefulness of a specific monitoring device in a clinical setting is also determined by the ease of its use and the amount of training required for the intensivist to obtain reliable data.

The objective of the study at hand was to assess the ability of novice operators with no previous experience in TEE to efficiently conduct hemodynamic assessments in an ICU setting. We systematically evaluated the image quality and inter-observer reliability during the introduction of the mTEE monitoring system in the ICU of a tertiary care center.

## Methods

This study is based on data collected during the prospective process quality audit in the context of the introduction of mTEE for clinical use in the Department for Intensive Care Medicine at the University Hospital of Bern, Switzerland and adheres to the tenets of the Declaration of Helsinki. The study protocol was submitted to the Ethical Committee of the Canton of Bern; the need for ethical approval was waived provided that purely observational data were collected in conjunction with the process quality audit. Nevertheless, all patients admitted to the Bern University Hospital are routinely informed of their right to specify whether data related to their stay can be used in observational studies; data of patients who declined were not included in the study.

The ImaCor ClariTEE^® ^probe is a miniaturized, 5.5 mm monoplane TEE probe (ClariTEE, ImaCor, Uniondale, NY, USA). The probe produces standard monoplane (transverse, 0-degree) two-dimensional images; anteflexion and retroflexion of the tip of the probe is possible. The probe is approved to remain *in situ *for 72 h. It is connected to a dedicated ultrasound system which allows for recording of digital loops and performance of basic two-dimensional measurements of areas and distances.

Data were collected between February 2012 and August 2012 for the first 55 patients in whom the device was used. Patient population included both medical and surgical patients. Only patients on mechanical ventilation with an orotracheal tube in place were examined. Ventilator settings routinely used in our department consist of volume controlled immediately after intubation and pressure support thereafter. Ventilator settings were not changed for the purpose of mTEE assessments; the department's standard sedation protocol was used to avoid patient-ventilator dyssynchrony. The exclusion criteria for the use of mTEE in our institution are as follows: unrepaired tracheoesophageal fistula, history of prior esophageal surgery, esophageal obstruction or stricture, esophageal varices or diverticulum, perforated hollow viscus, gastric or esophageal bleeding, vascular ring, aortic arch anomaly with or without airway compromise, oropharyngeal pathology, severe coagulopathy, and cervical spine injury or anomaly. The decision to use mTEE for hemodynamic monitoring was made by the treating staff specialist on clinical grounds.

Fourteen ICU staff specialists - none of whom had received any training in TEE before the study - received a total of six hours training in the use of mTEE by an experienced operator. Training included a 60-minute introduction of the method and demonstration of device use in the context of a presentation, followed by five hours of one-to-one bedside training in small groups of two to three trainees during the same day. Training included all necessary skills to use the mTEE device, and to acquire and interpret images according to predefined parameters. The mTEE examinations performed during the training period were not included in the analysis for the study at hand. At the time of the study, TEE examinations in our department were exclusively performed by an on-call cardiologist and not by intensive care staff; whereas 4 of the 14 operators assessed in the study had experience in TTE examination.

The decision to use mTEE for hemodynamic monitoring was at the discretion of the staff specialist responsible for the treatment of a patient and was not mandated by the study protocol. After positioning of the mTEE probe, three standard TEE views [11] were acquired, recorded and evaluated at the time of ICU admission or at the time of occurrence of hemodynamic instability by the ICU specialist in charge of the patients care: transgastric mid-esophageal short axis view, mid-esophageal four chamber view, and mid-esophageal ascending aortic short axis view. Left ventricular (LV) area at end-systole (LVESA) and at end-diastole (LVEDA) was measured from the transgastric mid-esophageal short axis view, the fractional area change (FAC) was calculated as LVEDA-LVESA/LVEDA and used to grade LV ejection fraction as normal (FAC >50%), moderately decreased (FAC 40 to 50%) or severely decreased (FAC <40%). Similarly, the ratio of right to left ventricular (RV) area was determined by measurements at end-diastole in the ME 4 chamber view. A ratio >0.6 was used as indicator of RV dysfunction [11]. The collapsibility of the superior vena cava was rated by calculating the collapsibility index, that is, the inspiratory decrease in superior vena cava diameter. The index was determined as (maximal diameter on expiration-minimal diameter on inspiration)/maximal diameter on expiration, expressed as a percentage. We used a threshold of >35% to distinguish between the presence and absence of hypovolemia [12].

After image acquisition the operator completed a questionnaire on the patient's hemodynamic condition based on the mTEE information. This included rating of systolic LV function (normal, moderately decreased or severely decreased), rating of RV size as dilated or not dilated, and presence or absence of hypovolemia and pericardial effusion. In addition, operators were asked to evaluate the difficulty to acquire the mTEE views (rated as easy, moderately difficult, difficult, not possible) and the utility of the information obtained by mTEE and to record if any changes in hemodynamic management (administration of additional fluids, dose adjustments of vasopressors/inotropes, drainage of pericardial effusion) were made on the basis of the acquired mTEE data. An independent observer (study nurse) recorded data during the mTEE examination regarding the successful use of the system and time from start of the system set-up at the bedside to completion of the questionnaire regarding the patient's hemodynamic condition.

All recorded mTEE views and measurements were assessed off-line by an expert cardiologist blinded to the patients and the mTEE operator's identity and to the results of the operator's examination. The mTEE views were assessed for adequate quality to measure FAC (transgastric mid-esophageal short axis view), RV/LV ratio (mid-esophageal four chamber view M) and superior vena cava collapsibility index (mid-esophageal ascending aortic short axis view). The cardiologist measured LV areas at end-systole and at end-diastole and calculated FAC for every patient and answered the same predefined questions about the hemodynamic condition based on the recorded mTEE views (systolic LV function normal - moderately decreased - severely decreased, RV dilatation present or absent, hypovolemia present or absent, significant pericardial effusion present or absent).

### Statistical analysis

Continuous variables are presented as mean (standard deviation) or median (interquartile range). Normality testing was performed using the Kolmogorov-Smirnov test. For comparison of mTEE measurements of ICU staff specialists and the cardiologist inter-rater reliability for categorical variables was assessed using a Chi-square test. Inter-rater agreement (kappa statistics) was determined. Pearson's r (1-tailed) was used to determine inter-rater reliability for continuous variables (FAC). A *P-*value of < 0.05 was considered statistically significant. Standard statistic software was used (IBM SPSS Statistics 19, Somers, NY 10589, USA).

## Results

### Patients

A total of 148 examinations were performed in 55 patients (mean age 63.7 ± 16.7 years, 59% male) during the study period, according for 2.7 ± 1.9 examinations per patient. Indication for mTEE assessment was circulatory failure in 45 patients (cardiogenic shock in 17 patients, septic shock in 17 patients, cardiac dysfunction post cardiac surgery in 8 patients, post resuscitation syndrome in 3 patients) and respiratory failure in 5 patients (3 patients pneumonia, 2 patients thoracic trauma) whereas 5 patients had other, miscellaneous indications. The mean duration of mTEE monitoring period in which the probe was left in place was 28 ± 16 hours.

### Examination feasibility and quality

**Figure 1:**
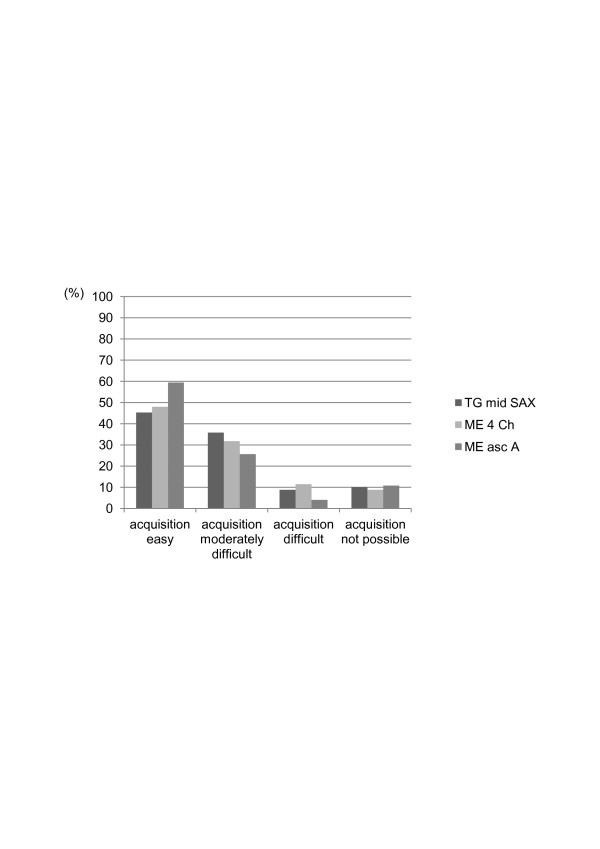
**Difficulty of acquiring mTEE views**. Difficulty of acquisition and image quality of three different transverse views obtained using the mTEE probe in 148 examinations of 55 patients with hemodynamic compromise. Difficulty of acquiring the views was rated by the ICU mTEE operator. ICU, intensive care unit; ME 4 chamber, mid-esophageal four chamber view; ME asc aortic SAX, mid-esophageal ascending aortic short axis view; mTEE, miniaturized trans-esophageal echocardiography; TG mid SAX, trans-gastric mid-esophageal short axis view.

All ICU staff specialists were able to use the system correctly and to store images and video loops after initial training. Median time from start-up of the device until completion of examination and recording of results was 23 minutes (IQR 15 to 28) if the mTEE probe was already in place and 25 minutes (IQR 20 to 33) if the mTEE probe had to be inserted. Insertion and/or acquisition of mTEE images required removal of the nasogastric tube in nine patients (16%) only for the initial placement and in six patients (11%), repeatedly accounting for the necessity of removal in 24 (16%) of all 148 examinations. Acquisition of the loops was considered easy in approximately half of the examinations and not possible in 9 to 10% of the examinations (Figure 1).

**Figure 2:**
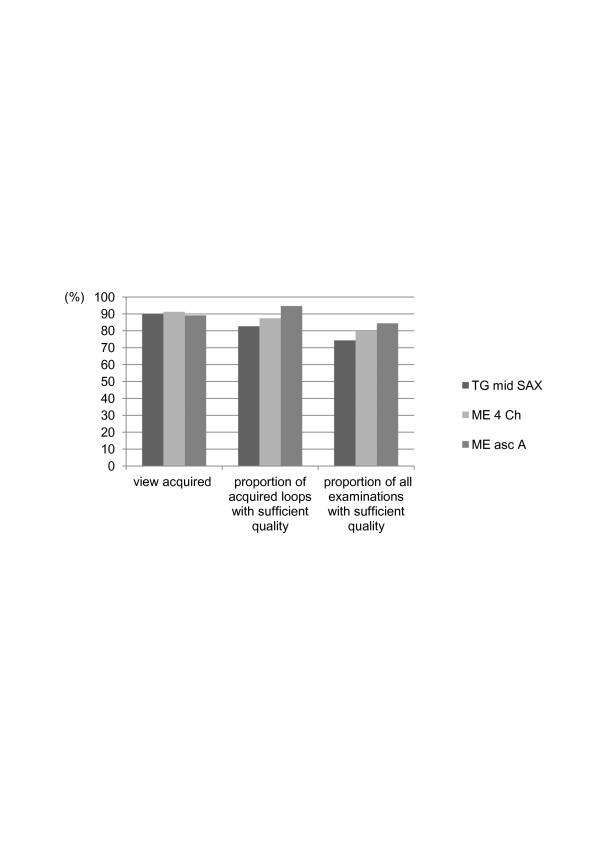
**Quality of mTEE views**. Percentage of all attempted views that were acquired stratified by the three standard mTEE views (TG mid SAX, ME 4 Chamber, ME asc aortic SAX) and proportion of all attempted loops and acquired loops with sufficient quality as assessed by a trained cardiologist. ME 4 chamber, midesophageal four chamber view; ME asc aortic SAX, mid-esophageal ascending aortic short axis view; mTEE, miniaturized trans-esophageal echocardiography; TG mid SAX, trans-gastric mid-esophageal short axis view

The quality of mTEE views was considered sufficient to assess the predefined hemodynamic parameters by the expert cardiologist in 110 examinations for trans-gastric mid-esophageal short axis, in 118 examinations for mid-esophageal four chamber and in 125 examinations for mid-esophageal ascending aortic short axis view (Figure 2). No complications due to mTEE use occurred during the study.

**Figure 3:**
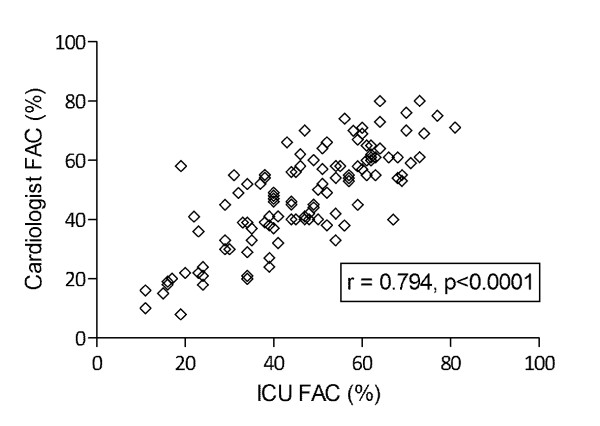
**Accuracy of measurements of left ventricular systolic function**. Assessment of the accuracy of measurements of left ventricular systolic function using the mTEE probe in 148 examinations of 55 patients with hemodynamic compromise. Measurements of left ventricular function fractional area change (FAC) by ICU operators were repeated by a trained cardiologist blinded to the patients and the mTEE operator's identity and to the results of the operator's examination. Correlation analysis revealed substantial inter-rater reliability of LV FAC measurements (*r *= 0.794, *P *(one-tailed) < 0.0001). ICU, intensive care unit; LV FAC, fractional area change of left ventricle; mTEE, miniaturized trans-esophageal echocardiography

Mean FAC was 47 ± 17%, and LVEF was described as normal in 45%, moderately decreased in 19% and severely decreased in 36% of examinations; RV-dilatation was present in 48% of the examinations, hypovolemia was detected in 5% and pericardial effusion was present in 23% of examinations. Inter-rater agreement between ICU mTEE operator and the expert cardiologist for estimates of LV function (Kappa 0.62, *P *< 0.0001), RV dilatation (Kappa 0.65, *P *< 0.0001) hypovolemia (Kappa 0.76, *P *< 0.0001) and occurrence of pericardial effusion (Kappa 0.77, *P *< 0.0001) was substantial. There was a significant correlation between the FAC measured by ICU operators and the cardiologist (*r *= 0.794, *P *(one-tailed) < 0.0001) (Figure 3).

Of the total of 148 mTEE assessments, the information acquired was rated as useful for the ongoing hemodynamic management of the respective patient in 113 (76%) examinations. A total of 56 changes in hemodynamic management after mTEE assessment of the respective patient were reported in 50 (34%) examinations. Management changes included administration of fluids in 19 instances (13%), start or increase of inotropes/vasopressors in 23 (16%), stop or decrease of inotropes/vasopressors in 10 (7%) and drainage of pericardial effusion following 4 examinations (3%).

## Discussion

Our study shows that the use of mTEE by operators not previously trained in TEE can be introduced successfully in an ICU setting of a tertiary hospital. Echocardiographic examinations by operators using mTEE after six-hour bedside training were technically feasible and of sufficient quality in a majority of examined ICU patients. Inter-rater reliability between assessment by ICU mTEE operators and a trained cardiologist was substantial. Hemodynamic assessment using mTEE might, therefore, provide a valuable alternative to standard TTE or TEE-examination or conventional hemodynamic monitoring for a rapid, semi-quantitative assessment of LV and RV function and volume status.

We evaluated a monoplane, oral mTEE probe for hemodynamic monitoring in a population of critically ill patients in our ICU. Our data show that after a short bed-side training our staff specialists were able to acquire and correctly interpret relevant information on key hemodynamic parameters. The acquired information was rated as useful for hemodynamic management in a majority of patients and led to therapeutic changes in a relevant proportion. No device specific complications occurred. The time needed for system start-up and to acquire the three mTEE views and process the images was substantial, but was comparable with the insertion of central lines for hemodynamic monitoring. Additionally, the duration is partially explained by the fact that in the context of the study we required the operators to assess and record all information in a protocolized fashion for every evaluation. In clinical practice, this is not necessary and real-time monitoring of hemodynamic parameters can be performed while the mTEE probe is left *in situ *for up to 72 hours. Our results are comparable with a recently published study by Vieillard-Baron *et al*. who evaluated the hemodynamic monitoring capability and safety of mTEE in a group of 94 ventilated critically ill patients [10]. The authors reported that a full hemodynamic evaluation (acquisition of all three mTEE views in optimal or acceptable quality) was possible in 85% of examinations. In their study 50% of hemodynamic assessments had a direct therapeutic impact. Fluid loading was performed in 41% patients, and inotropic or vasopressor support was initiated or increased in 33% and tapered off in 8% of patients.

Minor self-limited gastric bleeding mechanical ulceration of the superior lip was observed in two patients. However, mTEE examinations were performed by four highly trained intensivists with extensive expertise in critically care echocardiography. The authors state that their results cannot be extended to prove the ability of novice operators with no previous experience in TEE to efficiently conduct hemodynamic assessment using the tested device. Our results now also demonstrate that novice operators can obtain valid and clinically relevant hemodynamic data with mTEE although the success rate of obtaining images in sufficient quality was lower than the reported success rate of experienced TEE operators in the study of Vieillard-Baron *et al. *[10].

In an ICU setting, the ideal monitoring technique allows for fast and simple but accurate continuous measurement of key hemodynamic parameters, is of low invasiveness and does not require extensive training for its use [3]. Such a system does not currently exist but we must try and choose devices that have a maximum of these attributes and select the technique not only most appropriate for each patient, but also for each user. No hemodynamic monitoring improves patient outcome by itself. The quality of the obtained information, the correct interpretation of the data and the changes in management made as a result are of integral importance and have to be considered when assessing the clinical usefulness of novel monitoring modality.

Bedside echocardiography is considered a promising tool for the management of critically ill patients [13], and has been demonstrated to be useful in establishing the diagnosis and provides hemodynamic monitoring [14-16]. However, conventional TTE and TEE are not always readily available in an ICU setting. Additionally, TTE image quality is often impaired by factors such as sternotomies, chest drains, dressings or positive pressure ventilation leading to reported failure rates between 10 and 30% in different groups of ICU patients [17,18]. The approach of evaluating hemodynamic status by a simplified, time-sparing TEE examination for management of circulatory failure in septic shock has been successfully studied by others [19]. Nevertheless, a conventional TEE probe cannot be left in the patient for more than a few hours and repeated examinations are again time-consuming and may be associated with the increased risk of complications associated with the reinsertion [20]. The use of a transnasal, miniaturized TEE-probe has been shown to be feasible even in mildly sedated, intubated patients who were not fully cooperative and to provide information on cardiac function to the same extent as a conventional trans-oral monoplane TEE examination [21]. The transnasal probe seems to be better tolerated, but led to nasal bleeding in 31% of the study population. The main difference between the mTEE technology and a conventional TEE system is the possibility of leaving the probe in place for up to 72 h for repeated assessments and monitoring of the patient's condition. The use of the mTEE technology is limited by the lower resolution of the transducer and the lack of M-mode and pulsed wave and continuous wave Doppler. On the other hand, the disposable mTEE probe is detachable from the echo machine to allow the monitoring of several patients in a semi-continuous fashion with one device. How this new technology compares to other ultrasound-based or non-ultrasound-based monitoring modalities in terms of efficiency and efficacy remains to be studied.

Our study has several limitations. First, we did not compare the ability of a novice ICU operator with a TEE-trained cardiologist to obtain the images with the mTEE probe. It is conceivable that the high success rate could have been further improved by a trained expert. A study design allowing such comparison during hemodynamic instability could be hampered by relevant ongoing changes during the process of hemodynamic stabilization with fluids and vasopressors. We, therefore, chose to compare the interpretation of data off-line. Secondly, the study design allowed the use of any other, conventional hemodynamic monitoring modality at the discretion of the ICU specialist in charge of the patient. We cannot exclude that some of the reported decisions to change hemodynamic management also utilized additional available parameters rather than solely those acquired by mTEE - a practice we strongly advocate in the clinical routine of any hemodynamic monitoring. In our study, although hypovolemia was detected only in 5% of examinations, fluids were given nearly three times as often. We did not record the precise indication for the fluid administration, but assume that mTEE was also used to exclude dilated RV or LV when the aim was to enhance cardiac output or peripheral tissue perfusions with fluids. The impact on patient management was not the main outcome parameter of this feasibility study and we did not compare the effectiveness of mTEE monitoring with other monitoring technologies. Finally, it has to be emphasized that we report on the first mTEE examinations of each novice operator. The method of use in the context of the introduction of this new technology potentially does not reflect the future modalities of mTEE. Increasing experience might influence the decision processes for its use, such as patient selection and examination intervals, as well as technical properties such as time requirements for and quality of mTEE studies.

## Conclusions

In summary, our study shows that hemodynamic monitoring using the mTEE-system is feasible and provides additional, valid data for the management of critically ill patients after a brief training period of the operator. Further studies are required to assess the impact of hemodynamic monitoring by mTEE on relevant patient outcomes.

## Key messages

• Hemodynamic monitoring of ICU patients using the mTEE-system by operators not formally trained in TEE is feasible with minimal training.

• Hemodynamic monitoring of ICU patients using the mTEE-system provides additional, valid data for the management of critically ill patients.

• Further studies are required to assess the impact of hemodynamic monitoring by mTEE on relevant patient outcomes.

## Abbreviations

FAC: fractional area change; ICU: intensive care unit; LV: left ventricle; LVEDA: left ventricular end-diastolic area; LVESA: left ventricular end-systolic area; ME 4 chamber: mid-esophageal four chamber view; ME asc aortic SAX: mid-esophageal ascending aortic short axis view; (ClariTEE, ImaCor, Uniondale, NY, USA): mTEE: ImaCor ClariTEE^® ^miniaturized: 5.5 mm monoplane TEE probe; RV: right ventricle; TEE: trans-esophageal echocardiography; TG mid SAX: transgastric mid-esophageal short axis view; TTE: transthoracic echocardiography.

## Competing interests

The authors declare that they have no competing interests.

## Authors' contributions

TMM designed the study, performed the statistical analysis and drafted the manuscript. LC participated in the design of the study, assisted with the assessment of mTEE image quality and helped to draft the manuscript. HB independently assessed all mTEE examinations. DB and JW participated in the design of the study and assisted with the implementation of mTEE in our unit. JT conceived of the study, and participated in its design and coordination and helped to draft the manuscript. All authors revised the manuscript for important intellectual content; and have given final approval of the version to be published.
